# Solar-driven tandem photoredox nickel-catalysed cross-coupling using modified carbon nitride†

**DOI:** 10.1039/d0sc02131h

**Published:** 2020-06-25

**Authors:** Yangzhong Qin, Benjamin C. M. Martindale, Rui Sun, Adam J. Rieth, Daniel G. Nocera

**Affiliations:** Department of Chemistry and Chemical Biology, Harvard University 12 Oxford Street Cambridge Massachusetts 02138 USA dnocera@fas.harvard.edu

## Abstract

Nickel-catalysed aryl amination and etherification are driven with sunlight using a surface-modified carbon nitride to extend the absorption of the photocatalyst into a wide range of the visible region. In contrast to traditional homogeneous photochemical methodologies, the lower cost and higher recyclability of the metal-free photocatalyst, along with the use of readily available sunlight, provides an efficient and sustainable approach to promote nickel-catalysed cross-couplings.

## Abstract 2

Nickel-catalysed aryl amination and etherification are driven with sunlight using a surface-modified carbon nitride to extend the absorption of the photocatalyst such that they may be driven with solar light.

## Graphical abstract

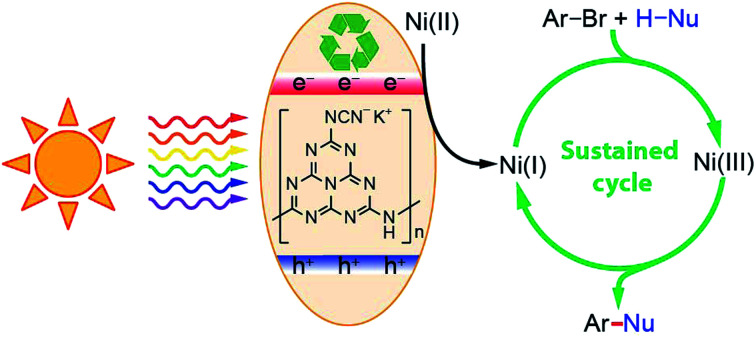

## Introduction

Photoredox chemistry has expanded the toolbox of chemical transformations owing to the ability to generate high energy and reactive radical intermediates with facility under mild conditions.^1–6^ Despite significant progress in the development of selective bond-breaking and -forming reactions resulting from photoredox-driven transformations using nickel, iron and copper complexes,^7–12^ many of these contemporary methods rely on homogeneous photocatalysts based on iridium or ruthenium, which are expensive and potential sources of toxic contaminants.^13–16^ Moreover, blue or UV light is often required to drive many photocatalytic reactions due to the limited absorption range of the common photocatalysts,^17–21^ which can result in off-pathway side reactions due to the competing excitation of other photoactive species present in solution. For instance, bipyridine-ligated nickel aryl halide complexes, often formed in nickel-catalysed cross-couplings, can absorb UV and blue light and result in unproductive homocoupling and dehalogenation.^22,23^

Although photoredox catalysis provides powerful strategies and new capabilities for synthesis, the cost of the photocatalyst and light source, as well as the environmental footprint of the synthetic method and catalyst recovery have been infrequently considered. To address this shortcoming, energy intensity (EI = total process energy/mass of final product) is an essential metric for quantifying the sustainability of chemical processes in addition to the more commonly used process mass intensity (PMI = mass of input material/mass of product),^24–26^ which only considers the materials input. For photocatalytic processes, energy and materials costs for generating photons, photocatalysts, and products can be accounted for by including EI with PMI. Additionally, costs are associated with removing toxic residues of the photocatalysts and their subsequent processing as chemical waste, the failure of which may result in detrimental consequences for the environment and public health.^27–29^ For example, the tolerable amount of metallic residues (*e.g.* platinum, iridium and ruthenium) in pharmaceuticals is strictly regulated. Therefore, strategies to drive chemical reactions with sustainable, recyclable and non-toxic materials are in high demand for improved energy efficiency, cost-effectiveness and minimal environmental impact.

Solar-driven chemical reactions are inherently sustainable and conducive towards a high EI.^3,30–32^ Although the power of sunlight at sea level is relatively high, on the order of 1000 W m^−2^ (based on ASTM G-173-03 standard), the energy is spectroscopically spread over a wide range from the UV region to the IR. Hence, an efficient photocatalyst that can maximally absorb broadband sunlight remains an unmet goal in photoredox catalysis. In contrast to homogeneous photocatalysts, semiconductors, such as GaAs and CdTe, can harness a broader wavelength of incident light; such materials appear to be promising photocatalysts for sustainable photocatalytic reactions.^33–36^ Nonetheless, the use of inorganic semiconductor colloids to harness sunlight and directly drive chemical reactions has remained challenging due to a variety of factors, including, but not limited to, surface passivation, charge recombination, materials instability and the balance between electron-transfer kinetics from the conduction and valence bands.^36,37^

**Scheme 1:**
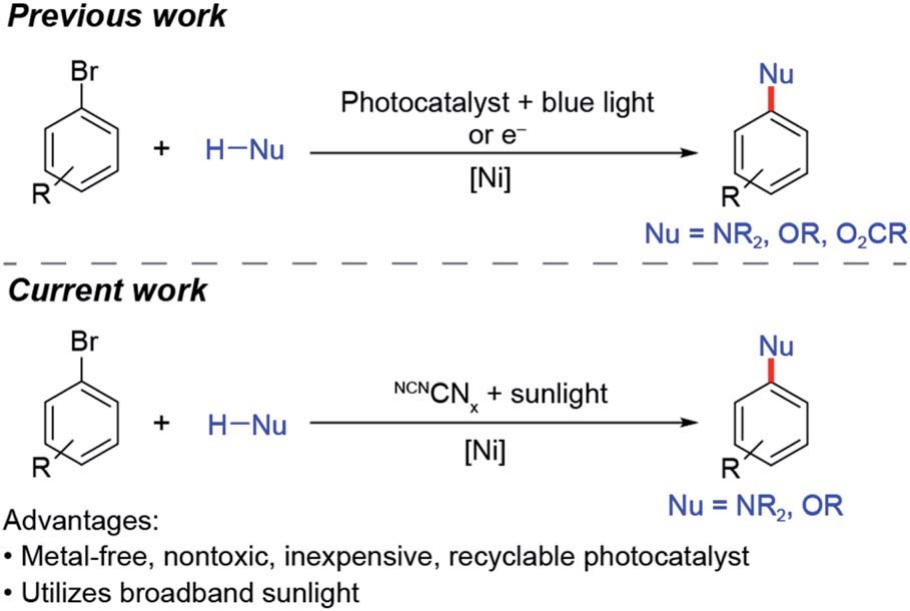
Summary of previous and current work on nickel-catalysed cross-coupling.

Cross-coupling between nucleophiles and aryl halides is a reaction that has employed both homogeneous and heterogeneous photocatalysts.^38–46^ To date, these reactions have required intense light sources and/or prolonged illumination.^1–6,17–21^ We now demonstrate that nickel-catalysed aryl amination (C–N) and etherification (C–O) may be directly driven by solar light using a surface-modified carbon nitride (Scheme 1), which extends absorption into a wide range of the visible region, thus making it amenable for solar photoredox catalysis. Additionally, besides being metal-free, the modified carbon nitride is nontoxic, inexpensive, easily synthesized and easily separated from product mixtures.

## Results and discussion

**Fig. 1:**
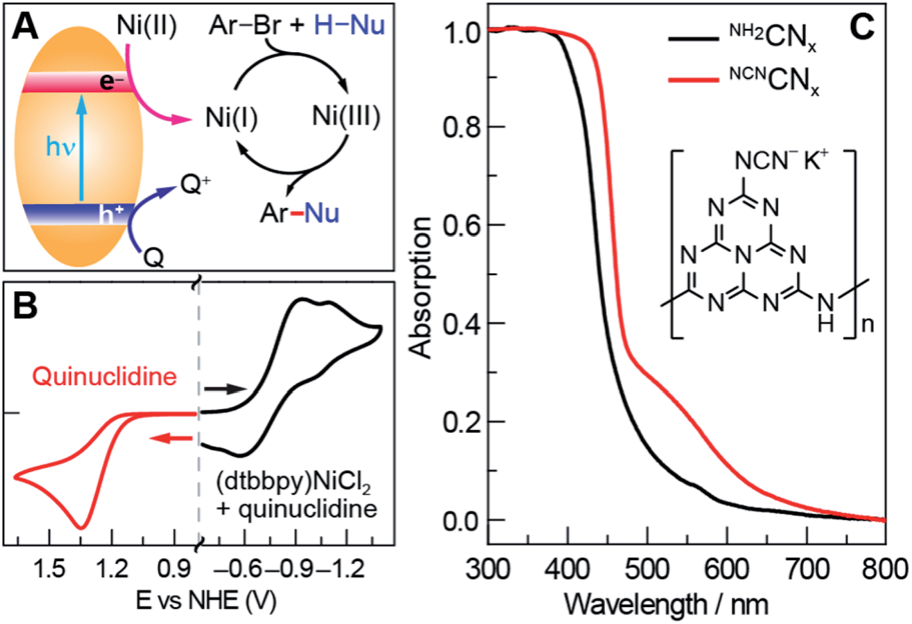
(A) A mechanistic scheme of the photoredox nickel-catalysed cross-coupling reaction. Q stands for quencher. (B) Cyclic voltammetry measurements of 2.5 mM (dtbbpy)NiCl_2_ in the presence of 5 mM quinuclidine, and 12 mM quinuclidine in acetonitrile. (C) Absorption spectra of unmodified carbon nitride (^NH_2_^CN_*x*_) and modified carbon nitride (^NCN^CN_*x*_). The inset shows a posited unit structure of ^NCN^CN_*x*_.

Photoredox nickel-catalysed aryl etherification was first reported with a polypyridyl iridium photocatalyst that is excited by blue LED light.^38^ The mechanism was further studied by our group and revealed to involve a sustained Ni(i/iii) dark cycle initiated by a one electron reduction of the Ni(ii) precatalyst (Fig. 1A).^47,48^ Significantly, our recent study shows that the photoredox reaction can also be realized by replacing the photocatalyst and photon source with a sub-stoichiometric amount of Zn metal,^48^ suggesting that the photocatalytic cycle serves as an electron source to return an off-cycle Ni(ii) complex to the active Ni(i) catalyst. This observation is consonant with the recent observation that Ni(ii) resides off-cycle in a resting state rather than as an on-cycle catalyst.^49^ These studies thoroughly explored the reaction conditions as well as the mechanism, which allowed us to employ optimized reaction conditions (see Table 1), and provide an underpinning for developing heterogeneous photocatalysts with attractive energy intensity.

**Table 1:** Testing C–O cross-coupling reactions with various heterogeneous photocatalysts^a^

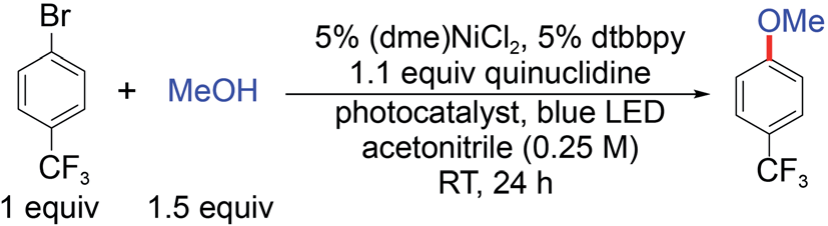				
Photocatalyst	Band gap (eV)	VB potential (V)	CB potential (V)	Yield (%)
SiC	3.0	1.5	−1.5	14
^NH^_^2^_CN_*x*_	2.7	1.8	−0.9	92
ZnSe	2.7	0.8	−1.9	0
GaP	2.3	1.2	−1.1	24
CdTe	1.4	0.7	−0.7	3

a Reactions were run on a 2 mL scale with 8 mg of photocatalyst. The band gap, valence band (VB) and conduction band (CB) potentials were taken from ref. 50–53, and all referenced to NHE at pH = 0.

A suite of heterogeneous catalysts comprising SiC, carbon nitride (^NH_2_^CN_*x*_), ZnSe, GaP and CdTe was surveyed with band gaps ranging from 3.0 eV to 1.4 eV.^50–53^Table 1 illustrates the efficacy of the photocatalysts for C–O coupling under illumination with 40 W blue LED light (Fig. S1†). The product yield was determined by ^1^H NMR spectroscopy using 1,3-benzodioxole as an internal standard. We found that ^NH_2_^CN_*x*_ gave the highest yield of product (92%) after 24 hours of illumination. ZnSe, possessing nearly the same band gap (2.7 eV) as ^NH_2_^CN_*x*_, yielded no product even though the conduction band is more reducing than that of ^NH_2_^CN_*x*_ (−1.9 V *vs.* −0.9 V as referenced to NHE at pH = 0, Table 1).^51,52^ Although they possess similar band gaps, the valence band potential of ZnSe is 0.9 V as compared to 1.8 V for ^NH_2_^CN_*x*_, suggesting that the less oxidizing valence band of ZnSe may prevent it from driving photocatalysis. Consistent with this contention, GaP and SiC, which have higher oxidation potentials for their valence bands (1.2 V and 1.5 V, respectively) than that of ZnSe, showed modest conversion yields of 24% and 14%, respectively, after 24 hours of illumination under blue light. To provide further insight as to the reaction disparity among heterogeneous photocatalysts, we examined, by cyclic voltammetry (CV), the reduction of the nickel complex, (dtbbpy)NiCl_2_ (dtbbpy = 4,4′-di-*tert*-butyl-2,2′-dipyridyl), in the presence of quinuclidine as the base, as well as the oxidation of the quinuclidine itself. Owing to the (quasi)irreversibility of the oxidation wave observed in CVs (Fig. 1B), a standard thermodynamic potential cannot easily be assigned for either species. However, the onsets of the nickel reduction wave at −0.6 V as well as the quinuclidine oxidation wave at 1.1 V are in line with our proposal that the potentials of the valence and conduction bands must be balanced to allow both cathodic and anodic processes to occur. Consequently, among all the heterogeneous photocatalysts tested, metal-free ^NH_2_^CN_*x*_ exhibits the best performance for C–O cross-coupling.

To utilize broadband sunlight, we next sought to extend the ^NH_2_^CN_*x*_ absorption by its chemical modification.^35,54,55^ Specifically, cyanamide-modified carbon nitride (^NCN^CN_*x*_) shows not only wider absorption in the visible region than that of ^NH_2_^CN_*x*_ (Fig. 1C)^56^ but also long-lived electrons in the conduction band, which cause the material to turn blue after reductive quenching of the hole upon photolysis.^57^ These long-lived electrons offer a continuous source of redox equivalents to reduce the Ni(ii) complexes and drive the productive Ni(i/iii) dark cycle. ^NCN^CN_*x*_ was synthesized from two inexpensive precursors (melamine and potassium thiocyanate) according to a previously reported procedure (see Section C in the ESI†).^56^ The absorption edge of ^NCN^CN_*x*_ is red-shifted compared to that of ^NH_2_^CN_*x*_, and the Tauc plot^58^ shows a minor change of 60 mV in the band gap (see Fig. S2†). This change is likely due to a slight displacement of the conduction band potential while maintaining the same valence band potential.^53^ Successful surface NCN functionalization was confirmed by Fourier transform infrared spectroscopy (FTIR) and X-ray photoelectron spectroscopy (XPS) (Fig. S3 and S4†), which showed a cyanamide stretch and the presence of potassium, respectively, consistent with previous reports.^56^ The modified carbon nitride (^NCN^CN_*x*_) performs better as a photocatalyst than ^NH_2_^CN_*x*_ under all irradiation conditions (Table S1†).

In analogy to aryl etherification, we posited that nickel-catalysed photoredox aryl amination could also proceed through a Ni(i/iii) dark cycle given the similarity in reaction conditions.^38,39^ A quantum yield in excess of unity (QY = 2.7 ± 0.1) was also reported for the C–N cross-coupling with [Ir(dF–CF_3_–ppy)_2_(dtbbpy)][PF_6_] as the photocatalyst,^48^ confirming the presence of a Ni(i/iii) cycle, analogous to the mechanism of aryl etherification. We note that, unlike aryl etherification catalysis, aryl amination does not require dtbbpy since the amine substrate can act as a ligand to stabilize the nickel centre.

**Fig. 2:**
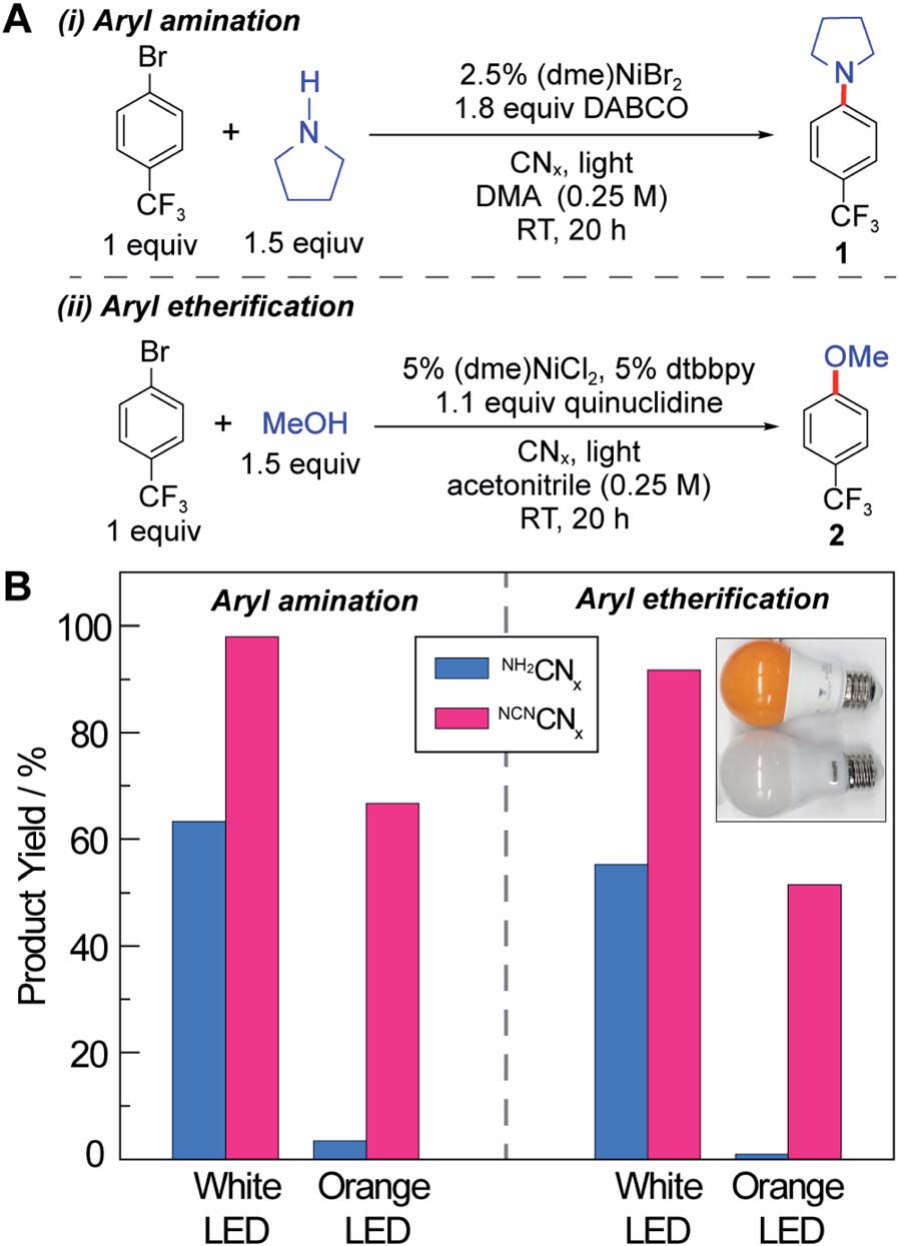
(A) Reaction conditions for nickel-catalysed aryl amination and etherification. (B) The product yield using photocatalyst ^NH_2_^CN_*x*_ or ^NCN^CN_*x*_ under illumination with either white or orange LED lamps.

Accurate quantum yield measurements are challenging for these heterogeneous reactions owing to scattering. Consequently, we performed external quantum yield measurements (EQY = number of product/number of incident photons) for both C–N and C–O cross-coupling (see Fig. 2A) using ^NCN^CN_*x*_ as the photocatalyst at low incident powers. Consistent with previous observations,^47,48^ we obtained EQYs in excess of unity (2.2 ± 0.2 and 2.4 ± 0.1 for C–N and C–O cross-coupling, respectively) under illumination with monochromic light at 435 nm (Section F.6 in the ESI†). EQY values in excess of unity unequivocally support the existence of dark cycle, as we have previously observed. Note that the internal quantum yields are higher because the incident photons in the EQY measurements are not all absorbed by the heterogeneous photocatalyst due to scattering.

The C–N and C–O coupling photocatalysis of the two carbon nitrides was examined using white LED and orange LED excitation (see the inset in Fig. 2B and Section E in the ESI†) under the reaction conditions shown in Fig. 2A, which are similar to the optimized conditions reported previously with homogeneous photocatalysts.^38,39^ For both cross-coupling reactions, ^NCN^CN_*x*_ outperforms ^NH_2_^CN_*x*_, especially as the excitation source is shifted into the red side of the visible absorption region. The white light LED has a spectrum peak at 460 nm in addition to a broadband emission centred at 600 nm, whereas the orange light only has the 600 nm band with no emission below 500 nm (see Fig. S1†). Notably, neither of the LEDs exhibit emission in the UV region. This is particularly important, because depending on the light intensity and the absorption spectrum of the photocatalyst, even a small portion of UV or blue light may be responsible for the majority of the observed activity in the presence of more intense but unproductive red light. For example, in a recent report on photoredox cross-coupling with carbon nitride,^43^ the white and green light sources both contained contributions from the UV region, which could be responsible for the observed reactivity based on the results reported here. After 20 hours of illumination under our UV-free white light, ^NH_2_^CN_*x*_ resulted in product yields of 63% and 55% for the C–N and C–O couplings, respectively, in contrast to the 98% and 92% product yields observed for ^NCN^CN_*x*_ (Fig. 2B). Moreover, after 20 hour illumination with the orange LED light source, ^NH_2_^CN_*x*_ only provided a trace amount of product (3% and 1%), whereas ^NCN^CN_*x*_ delivered 67% and 52% conversion for C–N and C–O coupling products, respectively. These results clearly demonstrate the enhanced photoactivity of ^NCN^CN_*x*_ under orange light due to its broad absorption band.

**Fig. 3:**
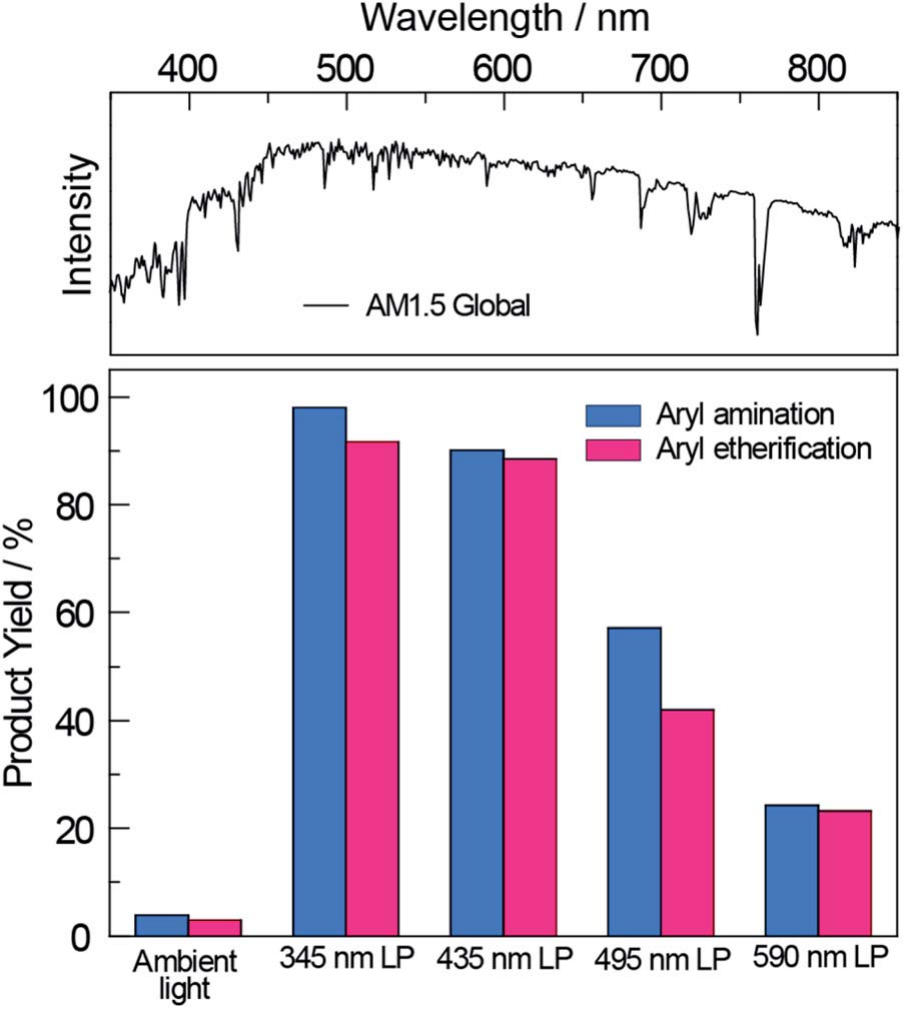
The product yield of nickel-catalysed aryl amination and etherification driven by broadband sunlight with ^NCN^CN_*x*_ as the photocatalyst. The reactions remain active even after applying a series of long pass filters with increasing cutting wavelength up to 590 nm. AM1.5 Global solar spectrum based on ASTM G-173-03 is shown at the top for reference.

Having demonstrated the improved photoactivity of the surface-modified carbon nitride in the visible region, the cross-coupling reactions were examined under one sun irradiance provided by a solar simulator (Newport Sol2A ABA class) with an AM1.5 filter (see Section E in the ESI†); a 345 nm long pass filter was employed to eliminate any deep UV light. As shown in Fig. 3, ^NCN^CN_*x*_ drove quantitative conversions for both cross-coupling reactions. By gradually red-shifting the cut-off wavelength of the long pass filter, we observed a decrease in the product yields commensurate with the diminished absorption of ^NCN^CN_*x*_. Nonetheless, even with a 590 nm long pass filter, product yields of 20% were achieved for both cross-couplings after 20 hours. These results are of particular significance since sunlight spans a broad spectrum where only a small fraction falls within the blue and UV region (see Fig. 3, top panel). Thus ^NCN^CN_*x*_ is able to harness an extended spectral range of sunlight (up to 590 nm) for photoredox cross-coupling reactions.

**Fig. 4:**
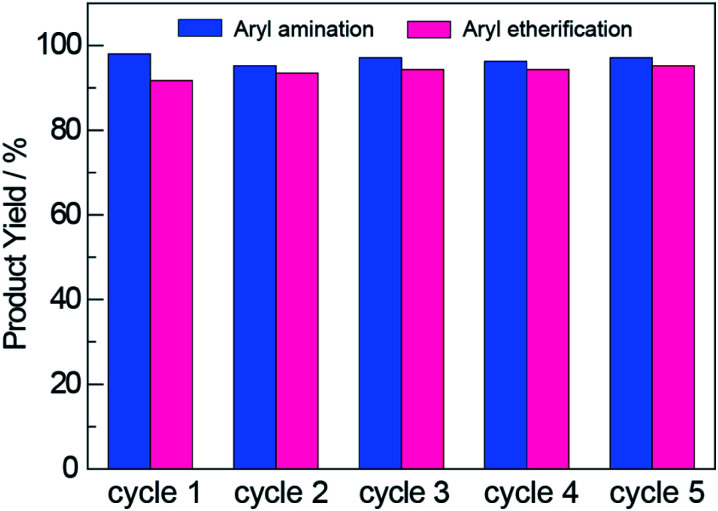
The ^NCN^CN_*x*_ photocatalyst was reused for multiple cycles over which no decrease in product yields was observed for either aryl amination or aryl etherification.

An additional advantage offered by heterogeneous photocatalysts is their facile recyclability, which is confirmed for ^NCN^CN_*x*_ in Fig. 4. The photocatalyst was recovered by subjecting it to three rounds of washing and centrifugation followed by drying at 130 °C in air (see Section D in the ESI†). The recovered ^NCN^CN_*x*_ showed no loss in activity after 5 cycles. Consistent with this result as well as those from previous reports^59,60^ that establish the NCN surface modification to be photostable, FTIR measurements showed that the NCN surface modification was retained with photocycling (Fig. S3†). To further investigate whether the activity observed using recycled photocatalyst was due to residual nickel deposited on ^NCN^CN_*x*_, we performed photoreactions with recycled ^NCN^CN_*x*_ (after 5 cycles) in the absence of Ni^2+^ complexes. For these control experiments, we observe 4% and 0% product yield for aryl amination and etherification, respectively. Consistent with previous reports,^46^ the results of these control experiments show that photocatalysis in recycling experiments is not due to nickel deposition on the ^NCN^CN_*x*_ photocatalyst.

**Fig. 5:**
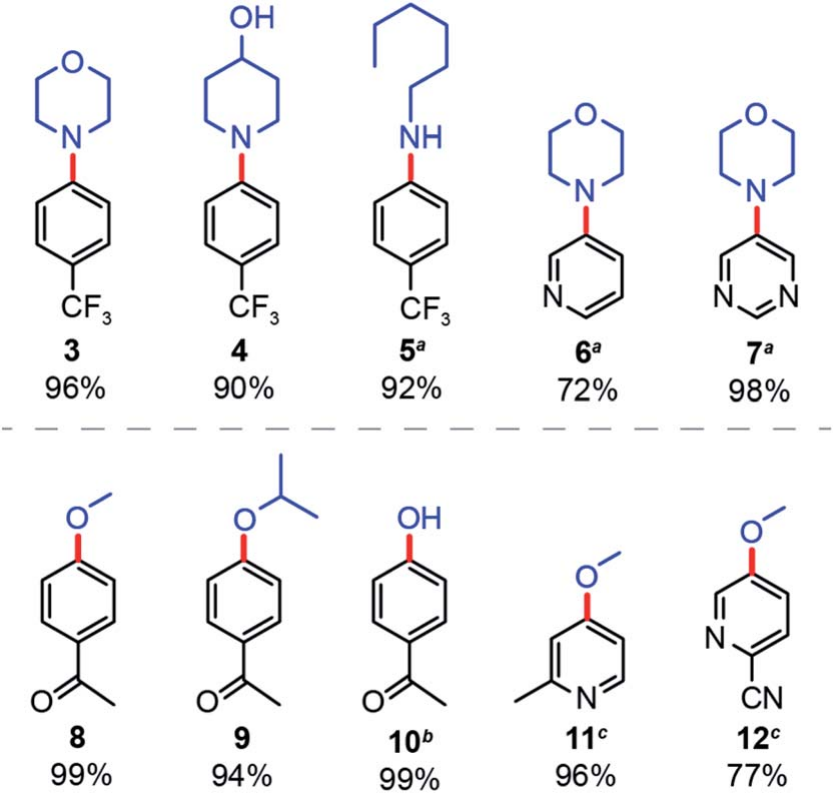
Applicability of nickel-catalysed aryl amination and etherification driven by sunlight and ^NCN^CN_*x*_. Yields were determined by ^1^H NMR signal referenced to 1,3-benzodioxole. ^*a*^Reaction heated to 55 °C. ^*b*^Different reaction condition, see Section B in the ESI.†^*c*^40 hours illumination.

Finally, the general applicability of ^NCN^CN_*x*_ was examined for the solar-driven nickel-catalysed cross-coupling between different aryl bromides and nucleophiles. For aryl amination (Fig. 5), we consistently obtained the expected products with high yields for various secondary (**3**, **4**) and primary (**5**) amines. Cross-coupling of heterocyclic aryl bromides proved to be more challenging,^39^ but heating to 55 °C furnished product in good yields (**6**, **7**). Similarly, for aryl etherification, methanol and isopropanol furnished product yields of over 90% (**8**, **9**). Surprisingly, water can be cross-coupled with 99% yield under slightly modified conditions (**10**) (see Section B in ESI†). Heterocyclic aryl bromides were also coupled successfully in good yields with a reaction time of 40 hours (**11**, **12**).

## Conclusions

We have demonstrated that surface-modified carbon nitride, ^NCN^CN_*x*_, with its extended absorption in the visible region, can directly utilize broadband sunlight to drive nickel-catalysed aryl amination and etherification. In contrast to UV- and blue-light driven photoredox processes, the method disclosed herein enables highly efficient bond formation at high energy intensity (EI) while avoiding competing photoreactions of other chemical species frequently encountered in dual photocatalytic processes. Owing to its increased efficiency in solar light absorption, metal-free nature, facile separation, ready recyclability, and straightforward synthesis, ^NCN^CN_*x*_ is an attractive heterogeneous photocatalyst for environmentally benign and sustainable solar synthesis.

## Conflicts of interest

There are no conflicts to declare.

## Supplementary Material

SC-011-D0SC02131H-s001
