# Supplementary material for: Solar-driven tandem photoredox nickel-catalysed cross-coupling using modified carbon nitride
Source: Chem Sci. 2020 Jun 25;11(28):7456–61. doi: 10.1039/d0sc02131h (PMC8159281; doi:10.1039/d0sc02131h)
Supplement: SC-011-D0SC02131H-s001 [file SC-011-D0SC02131H-s001.pdf]

Electronic Supplementary Material (ESI) for Chemical Science.
This journal is © The Royal Society of Chemistry 2020

Supporting Information

Solar-driven tandem photoredox nickel-catalysed cross-coupling using a modified carbon nitride

Yangzhong Qin,[†] Benjamin C. M. Martindale,[†] Rui Sun, Adam J. Rieth and Daniel G. Nocera*

Department of Chemistry and Chemical Biology, Harvard University, 12 Oxford Street, Cambridge, MA 02138, USA

*Email: dnocera@fas.harvard.edu

†These authors contribute equally

Table of Contents

A. General Considerations 3

B. Photoredox Reaction Solutions 3

C. Carbon Nitride Preparation 3

D. Carbon Nitride Recycling 4

E. Photocatalytic Reactions 4

F. Measurements 5

F.1 UV-vis Absorption and Emission Spectroscopy 5

F.2 FTIR Spectroscopy 7

F.3 X-Ray Photoelectron Spectroscopy 8

F.4 NMR Spectroscopy 9

F.5 Cyclic Voltammetry 9

F.6 External Quantum Yield Measurement 9

G. References 10

A. General Considerations

All reagents were purchased from commercial suppliers and used without further purification. All non-deuterated solvents were purified by an argon purged solvent purification system and stored over activated 3 Å molecular sieves in a N_2-filled glovebox. Semiconductors (SiC, ZnSe, GaP and CdTe) were purchased from Sigma Aldrich and those that were not powders were ground with an agate mortar and pestle prior to use. All reaction solution preparations were performed in a N_2-filled glovebox unless otherwise stated.

B. Photoredox Reaction Solutions

For aryl amination, 0.5 mmol aryl bromide (1 equiv), 0.75 mmol amine (1.5 equiv), 12.5 μmol (dme)$NiBr_2$ (0.025 equiv), 0.9 mmol DABCO (1.8 equiv) and 0.05 mmol 1,3-benzodioxole (0.1 equiv) were placed into a 20-mL glass vial and dissolved in 2 mL of DMA as the solvent. For aryl etherification, 0.5 mmol aryl bromide (1 equiv), 0.75 mmol alcohol (1.5 equiv), 25 μmol (dme)$NiCl_2$ (0.05 equiv), 0.55 mmol quinuclidine (1.1 equiv), 25 μmol dtbbpy (0.05 equiv) and 0.05 mmol 1,3-benzodioxole (0.1 equiv) were placed into a 20-mL glass vial and dissolved in 2 mL of acetonitrile as the solvent. The solution was stirred for 30 min on a magnetic stirrer and filtered through a 0.2-μm PTFE syringe filter to furnish a clear solution which was then stored in a 20-mL vial. 8 mg (4 mg/mL) carbon nitride powder was subsequently added to the solution. All these procedures were performed in a N_2-filled glovebox. Finally, the vial was taken out of the glovebox and immediately sealed with vinyl electric tape before irradiation.

For the cross-coupling reaction between water and 4'-bromoacetophenone, 0.4 mmol 4'-bromoacetophenone (1 equiv), 0.80 mmol *N*-*tert*-butylisopropylamine (2 equiv), 0.02 mmol (diglyme)$NiBr_2$ (0.05 equiv), 0.02 mmol dtbbpy (0.05 equiv) and 0.05 mmol 1,3-benzodioxole (0.125 equiv) were placed in a 20-mL glass vial and dissolved in 2 mL of DMF as the solvent. The solution was stirred for 30 min and then filtered through a 0.2-μm PTFE syringe filter to yield a clear solution. Lastly, 4 mmol water (10 equiv) and 8 mg carbon nitride were added to make the final reaction mixture.

C. Carbon Nitride Preparation

Both $^{NH_2}CN_x$ and $^{NCN}CN_x$ were prepared based on a published procedure.[1] $^{NH_2}CN_x$ was prepared by heating melamine to 550 °C for 4 h under Ar and the resulting polymer was ground into a powder using an agate pestle and mortar. To prepare $^{NCN}CN_x$, the $^{NH_2}CN_x$ powder was mixed with dried KSCN in a 1:2 weight ratio. The mixture was then heated to 400 °C for 1 h and 500 °C for 0.5 h under Ar. After cooling, the polymer was ground into a powder again with a pestle and mortar. The residual KSCN was washed away by subjecting the powder to a large amount of DI water and vacuum filtration using a paper filter (Whatman 1001-070). Finally, the compound was dried at 60 °C under vacuum and ground again to furnish $^{NCN}CN_x$ powder.

D. Carbon Nitride Recycling

When each round of the reactions was completed, the reaction solutions in their original 20-mL reaction vials were centrifuged at 4400 rpm for 45 min (Eppendorf centrifuge 5702 R). A 0.1 mL aliquot of solution was then taken from the top for product yield measurement. The rest of the solution was mixed with ~20 mL acetone and shaken vigorously to re-suspend the carbon nitride. The mixture was centrifuged again at 4400 rpm for 45 min and the top clear solution was disposed. This completes the first cycle of the "washing" process. Two subsequent "washings" with ~20 mL acetone and ~20 mL ethanol were performed. After that the resulting "wet" carbon nitride was dried in an oven at 140 °C overnight. The dry recycled carbon nitride was brought into the glovebox and recharged with fresh reaction solution for the next reaction cycle.

E. Photocatalytic Reactions

To screen the heterogeneous photocatalysts (Table 1), we tested the aryl etherification reaction by placing reaction samples (maximum 2) 5 cm from a single 40 W blue light source (Kessil A160WE Tuna Blue). To compare the activity of $^{NH_2}CN_x$ and $^{NCN}CN_x$, we tested the same reaction (as shown in Table S1) with the two reaction samples situated between two identical light sources, which were two 9.5 W soft white LED A19 bulbs (Philips) or two 9 W orange LED A19 bulbs (Triglow). The distance between the edge of the sample vial and the surface of the two light bulbs was about 2 cm. For the solar-driven photoreactions, excitation was provided by a Newport Sol2A ABA class solar simulator equipped with an AM1.5 filter. The reaction samples (typically 2) were

Table S1. Photoredox aryl etherification under various conditions.

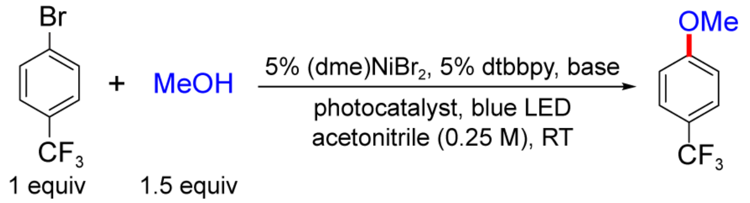

Photocatalyst	Photocatalyst Loading (mg/mL)	Reaction Time (h)	Base (equiv)	Yield (%)
$^{NH_2}CN_x$	4	24	1.1, Quin	92
$^{NCN}CN_x$	4	24	1.1, Quin	99
$^{NH_2}CN_x$	2	24	1.1, Quin	76
$^{NCN}CN_x$	2	24	1.1, Quin	98
$^{NH_2}CN_x$	2	2	1.1, Quin	23
$^{NCN}CN_x$	2	2	1.1, Quin	72
$^{NCN}CN_x$	4	24	3.0, TEA	46
$^{NCN}CN_x$	4	24	1.5, TEA	37
$^{NCN}CN_x$	4	24	1.5, DBU	17

Quin = Quinuclidine; TEA = triethylamine; DBU = 1,8-diazabicyclo[5.4.0]undec-7-ene. Yield was determined based on ^1H NMR signal referenced to 1,3-benzodioxole.

placed at a distance at which the light intensity was equal to one sun irradiance, as measured by a Newport 91150V solar reference cell and meter. A 5 × 5 in^2 filter holder fabricated by 3D printing was placed before the reaction samples so that only filtered sunlight could reach the sample. All photoreactions were run under constant stirring by a magnetic stirrer. For room temperature reactions, a high-speed fan was used to cool the sample. For reactions run at elevated temperature, the reactions samples were placed on a hotplate equipped with a thermocouple.

F. Measurements

F.1 UV-Vis Absorption and Emission Spectroscopy

The emission spectra of different lights sources (Figure S1) were measured by a CCD Array UV-vis spectrometer (SI Photonics, USA) running in the intensity mode. The measurement was carried out in a dark room. The diffuse reflectance spectra were measured with a Varian Cary 5000 UV-vis-NIR spectrometer equipped with a Praying Mantis diffuse reflection accessory (Harrick Scientific Products). The absorption spectra were constructed based on Abs = (R_{ref} – R_{sample})/R_{ref}, where R_{ref} and R_{sample} stands for the reflectance spectra of the reference and sample, which are PTFE powder and sample/PTFE mixed powder, respectively.

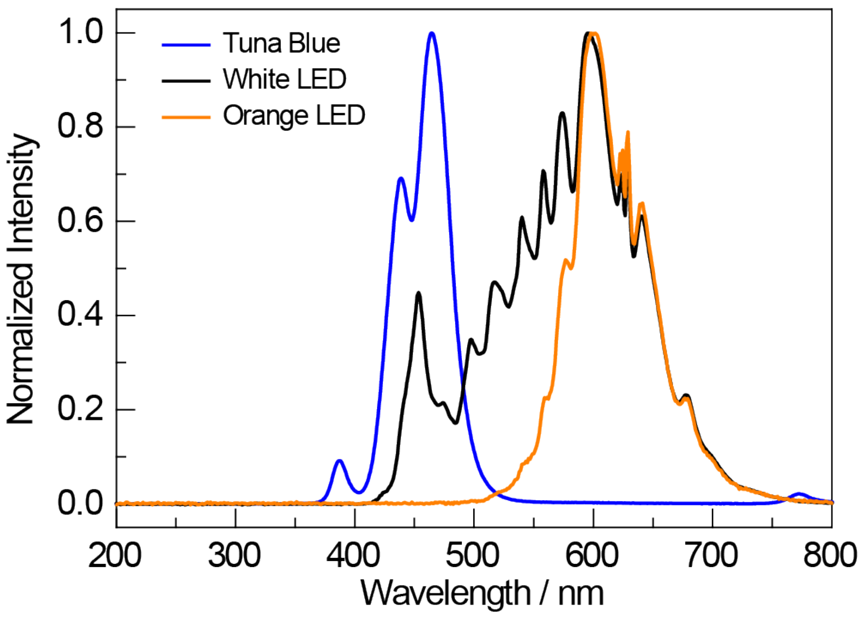

Figure S1. The emission spectra of different light sources including the Kessil A160WE tuna blue lamp (40 W), Philips soft white LED A19 bulb (9.5 W) and Triglow orange LED A19 bulb (9 W).

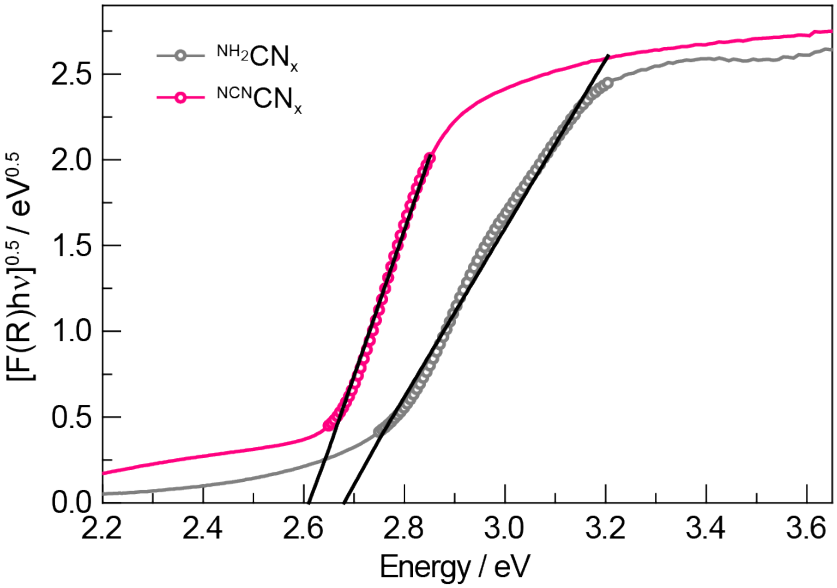

Figure S2. Tauc plot based on the diffuse reflectance, R, and the Kubelka-Munk function F(R). Selected data marked by the scattered points were fit linearly (black and red lines) to yield a band gap of 2.68 eV and 2.62 eV for $^{NH_2}CN_x$ and $^{NCN}CN_x$, respectively.

F.2 FTIR Spectroscopy

FT-IR spectra were taken on dry powder samples using a PerkinElmer Spectrum 400 FT-IR Spectrometer.

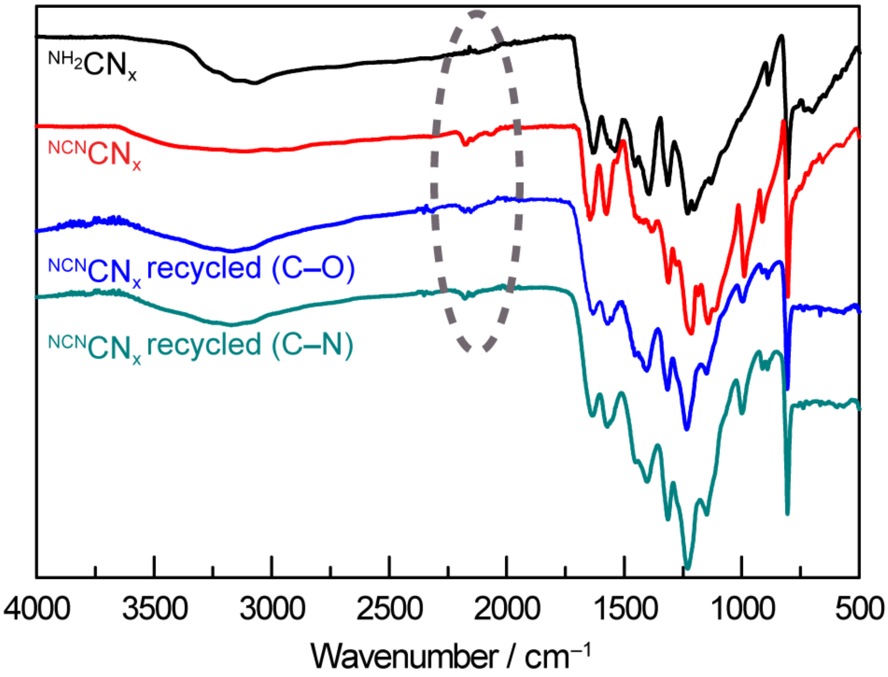

Figure S3. FTIR spectra for carbon nitride. Spectra for freshly prepared $^{NH_2}CN_x$ and $^{NCN}CN_x$ are shown in the black and red traces, respectively. Spectra for recycled $^{NCN}CN_x$ after 5 cycles of C–O and C–N coupling reactions are shown in blue and dark cyan traces, respectively. The surface modification for $^{NCN}CN_x$ is shown by the C≡N stretching band at 2177 cm^{-1} (dashed oval region), which remains after 5 cycles of reaction, indicating the photostability of the NCN surface modification.

F.3 X-Ray Photoelectron Spectroscopy

X-ray photoelectron spectroscopy (XPS) was performed at the Harvard Center for Nanoscale Systems (Cambridge, MA, USA) on a Thermo Scientific K-Alpha+ system equipped with an Al source and 180° double focusing hemispherical analyzer and 128-channel detector using a 30 µm X-ray spot size. Dry powder samples were mounted on two-sided tape on aluminum foil substrates.

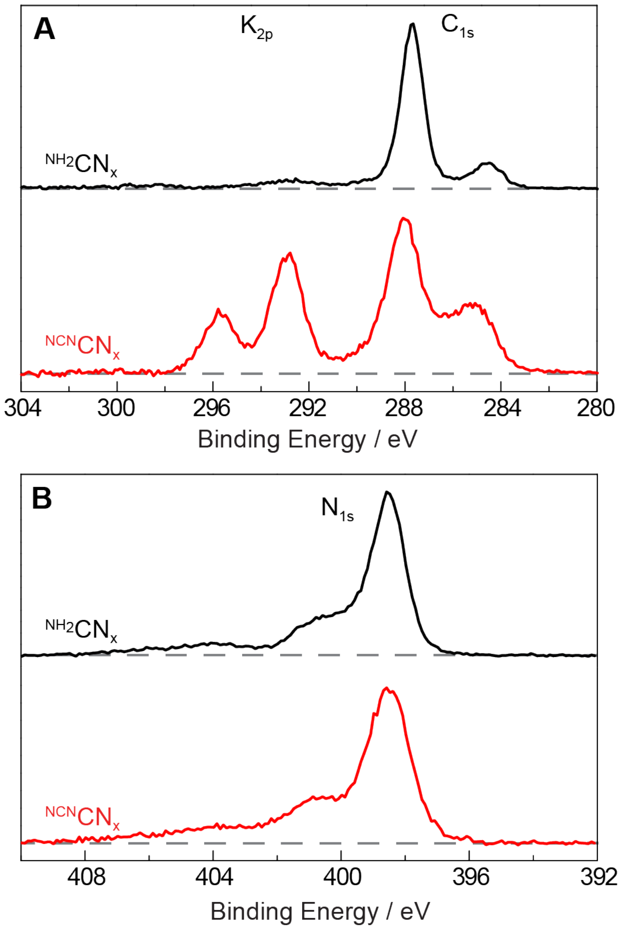

Figure S4. XPS spectra for $^{NH2}CN_x$ and $^{NCN}CN_x$ in the region of (**A**) C_{1s} and K_{2p} (**B**) and N_{1s}. The K_{2p} signal (290-298 eV) suggests successful surface modification $^{NCN}CN_x$. The N_{1s} signal was also consistent with previous observation.[1]

F.4 NMR Spectroscopy

NMR spectra were recorded at the NMR facility located at the Harvard University Department of Chemistry and Chemical Biology. ^{1}H or ^{19}F spectra were collected on an Agilent DD2 spectrometer (600 MHz) or a Varian/Inova spectrometer (500 MHz). The reaction solutions were made with proteo-solvents (DMA, acetonitrile or DMF) containing pre-quantified 1,3-benzodioxole as the internal standard. To prepare the NMR samples, a 0.1 mL aliquot of reaction solution was mixed with 0.8 mL $CDCl_3$, and the solution was filtered and then placed in an NMR tube.

F.5 Cyclic Voltammetry

Cyclic voltammetry (CV) measurements were carried out with a CH Instruments (CHI) potentiostat 760D and Version 10.03 software in a nitrogen filled glovebox. The compounds were dissolved in either acetonitrile or DMA with 0.1 M n-Bu_4NBF_4 as the electrolyte. A three-electrode configuration was used with a glassy carbon working electrode, Pt wire counter electrode and non-aqueous Ag/Ag^+ reference electrode. All glassy carbon working electrodes were polished on felt using 3-µm and 1-µm diamond pastes before use. The CV of a 1 mM ferrocene (Fc) solution was taken at the beginning of each experiment as a reference.

F.6 External Quantum Yield Measurement

The external quantum yield (EQY) was measured for both C–N and C–O cross-couplings (see Figure 2A in the main text) using the following formula,

$$EQY = \frac{number\ of\ product\ molecule}{number\ of\ incident\ photon} = \frac{cV}{\emptyset t}$$

where c is concentration of product, V is the volume of the reaction solution, $\emptyset$ is the photon flux and t is the reaction time. For each measurement, 1.5 mL of solution (see Section B) and 6 mg $^{NCN}CN_x$ were placed in a 1 cm path length cuvette and illuminated by monochromic light provided by a 150 W Xe arc lamp (Newport 67005 arc lamp housing and 69907 power supply) and a 435 nm band pass filter (FWHM = 10 nm). The light was focused onto the sample by a focusing lens (f = 40 mm). The power was adjusted by a neutral density filter and measured by an Ophir ORION/PD power meter and PD-300-ROHS head sensor. The photon flux was further calibrated to be 4.21×10^{9} mol/s (corresponding to a power of 1.16 mW) by ferrioxalate based on a published procedure.[2] The product concentration was determined by ^{1}H NMR spectrum referenced to 1,3-benzodioxole as an internal standard. The reactions were run three times to furnish an average quantum yield and standard deviation.

G. References

(1) Lau, V. W.; Moudrakovski, I.; Botari, T.; Weinberger, S.; Mesch, M. B.; Duppel, V.; Senker, J.; Blum, V.; Lotsch, B. V. Rational Design of Carbon Nitride Photocatalysts by Identification of Cyanamide Defects as Catalytically Relevant Sites. *Nat. Commun.* **2016**, *7*, 12165.

(2) Hatchard, C. G.; Parker, C. A. A New Sensitive Chemical Actinometer-II. Potassium Ferrioxalate as a Standard Chemical Actinometer. *Proc. R. Soc. London, Ser. A* **1956**, *235*, 518-536.
